# Evaluating a Digital Intervention to Reduce Aggression and Pro-Firearm Violence Attitudes Among Young Black Males: Pretest-Posttest Feasibility Study

**DOI:** 10.2196/70048

**Published:** 2025-08-25

**Authors:** Chuka Emezue, Jessica Bishop-Royse, Andrew Froilan, Tara Wilkes, Niranjan S Karnik, Wrenetha A Julion

**Affiliations:** 1 Department of Women, Children and Family Nursing, College of Nursing, Rush University, 600 S Paulina St, Suite 1080, Armour Academic Center, Chicago, IL, 60612, United States, 1 3129426151; 2 Department of Psychiatry, University of Illinois Chicago, College of Medicine, Institute for Juvenile Research, Chicago, IL, United States

**Keywords:** mental health, youth violence, substance use, digital intervention, prevention

## Abstract

**Background:**

Pediatric and adolescent firearm injuries and fatalities in the United States have surged to levels not seen since the mid-1990s, marking a critical public health inflection point. Young Black males (ages 15‐24) experience firearm-related fatality rates 24 times higher than their White peers. Despite this disproportionate risk, they are less likely to participate in traditional firearm violence prevention programs. This disparity highlights the urgent need for innovative, culturally responsive approaches that address the emotional, behavioral, and social determinants of violence.

**Objective:**

This pilot study aims to evaluate the preliminary effects of BrotherlyACT, a culturally responsive, trauma-informed, multicomponent mobile and web-based intervention designed to support young Black males (ages 15‐24) in navigating and preventing community violence, substance use, and mental health challenges. The intervention aims to increase access to precrisis support and mental health resources for youth living in low-resource, high-violence settings.

**Methods:**

Seventy young Black males with Serious Fighting, Friend Weapon Carrying, Community Environment, and Firearm Threats (SaFETy) scores between 1 and 5 (indicating low-to-moderate firearm violence risk) were enrolled in this prospective pretest-posttest study. Participants completed a psychoeducational component of the BrotherlyACT intervention, consisting of 7 video-based modules. Surveys were administered at baseline and again 4 weeks later to assess changes in attitudes toward guns and violence (Attitudes Toward Guns and Violence Questionnaire), reactive and proactive aggression (Reactive-Proactive Aggression Questionnaire), psychological distress (Kessler Psychological Distress Scale), and depressive symptoms (8-item Patient Health Questionnaire). Paired *t* tests were conducted to analyze pre-post differences.

**Results:**

A total of 70 young Black males (mean age 20.97 years, SD 2.44 years) participated in the study. Nearly half reported recent physical fights (48/70, 69%), gun threats (39/70, 56%), or hearing gunshots in their neighborhood (63/70, 90%). More than 50% (39/70, 56%) reported illicit drug use, and 32 out of 70 (46%) reported substance-related violence. SaFETy scores revealed heterogeneous but elevated exposure to firearm risk factors, particularly in community violence and firearm threats. Postintervention, participants demonstrated a statistically significant reduction in attitudes toward guns and violence (Attitudes Toward Guns and Violence Questionnaire; mean 29.8-26.1, *P*<.001, *d*=0.53), with the largest shift observed in “Aggressive Response to Shame” (28% reduction). Reactive aggression significantly declined (mean 10.48-8.67, *P*=.008, *d*=0.37), whereas proactive aggression remained stable. Psychological distress and depressive symptoms remained stable. Nearly all participants (68/70, 97%) completed all modules in a single session, with 47 out of 70 (67%) finishing within an hour, suggesting high feasibility and user engagement.

**Conclusions:**

Preliminary findings indicate that BrotherlyACT may reduce proviolence attitudes and reactive aggression among young Black males. These results underscore the feasibility and potential impact of culturally responsive digital interventions as a strategy to prevent firearm violence among underserved youth populations.

## Introduction

Firearm injuries and homicides constitute a national public health crisis in the United States, representing the leading cause of death among children and youth aged 0‐24 years [1,2]. This epidemic, including firearm homicides, suicides, incidental shootings, and nonfatal incidents, disproportionately affects children and adolescents in low-income urban and rural communities. Young Black males aged 15–24 experience the highest firearm homicide burden in the United States, with rates reaching 52.9 per 100,000 [2-4], more than 18 to 24 times higher than those of their White male peers, particularly in the aftermath of the COVID-19 pandemic [3,5-9].

Black youth remain historically underserved in mental health and early violence prevention systems (ie, primary prevention). Barriers to accessing trauma-informed care include stigma, institutional mistrust, racialized criminalization, and systemic underinvestment in their communities [10]. Even after injury, long wait times, limited follow-up, cold referrals, and provider mistrust deter intervention and engagement with follow-up services [11-16]. Critically, a major limitation of existing pediatric firearm injury prevention programs is their focus on postviolence contexts, typically after youth present in emergency departments (EDs) [17-19] or at advanced stages of violence involvement, such as during adjudication or institutionalization [16,17,20]. While ED-based programs show promising effects in reducing aggression and trauma [17,18,21-23], they face challenges such as staffing shortages, reimbursement issues, and difficulties with postdischarge follow-up [18]. These systemic and structural barriers continue to discourage young male engagement in interventions and contribute to service avoidance or early dropouts from programs.

Before an injury or ED contact, psychosocial and behavioral risk factors, such as aggression, emerge as modifiable determinants of firearm violence that can be addressed early in life [3,7,24-28]. Within this context, 2 subtypes of aggression are noteworthy: reactive and proactive aggression. Reactive aggression is characterized by impulsive responses to perceived threats and emotional dysregulation [29-32], whereas proactive aggression is calculated, planned, and self-serving, often associated with status and protection [33,34]. These aggression subtypes predict firearm risk more reliably than household firearm access alone [35], yet they are rarely targeted in primary prevention efforts aimed at reducing pediatric firearm injuries and homicides.

Recent evidence indicates that digital mental health interventions can effectively improve mental and behavioral health outcomes among children and adolescents, including those from high-risk or marginalized backgrounds with limited access to existing and traditional resources. In a systematic review focused on low- and middle-income settings, Lehtimaki et al [36] found that digital interventions produced promising reductions in adolescent depression and anxiety. Similarly, self-guided digital interventions have been shown to reduce suicidal ideation among youth [37]. In high-income contexts, Lattie et al [38] emphasized the importance of user-centered design in mobile and web-based tools targeting depression and anxiety among young adults. Additionally, Ranney et al [39,40] demonstrated that app-based tools used in ED settings can reduce aggressive behaviors in urban adolescents. These findings highlight the potential of digital health interventions as scalable and accessible tools for youth mental health and violence prevention.

Building on this evidence, we developed *BrotherlyACT*, a culturally responsive, trauma-informed, multicomponent mobile and online intervention designed to support young Black males (aged 15‐24 years) in navigating and preventing community violence, substance use, and mental health challenges. The app aims to improve access to precrisis support and mental health resources for youth living in low-resource, high-violence environments. We conducted a single-group, prospective pre-post pilot study to evaluate the preliminary effects of BrotherlyACT’s video-based psychoeducational modules. Primary outcomes were changes in reactive and proactive aggression, firearm-related attitudes, and mental health indicators.

## Methods

### Study Design

This prospective pretest/posttest pilot study was conducted from June to July 2024. It adheres to the guidelines for nonrandomized pilot and feasibility studies, as well as the CONSORT-EHEALTH (Consolidated Standards of Reporting Trials of Electronic and Mobile Health Applications and Online Telehealth) guidelines. A comprehensive description of the study protocol is provided elsewhere [41].

In addition, prior usability testing and heuristic evaluation of the BrotherlyACT intervention involving young Black males and mobile health (mHealth) specialists have been documented elsewhere [42]. The app received an average System Usability Scale score of 79, equivalent to an A-minus grade and placing it in the 85th percentile, indicating near-excellent usability [42]. Similarly, heuristic evaluation by mHealth experts identified only minor and cosmetic usability issues, with a median severity score of 1 across various heuristics (on a scale of 0‐3), indicating minimal impact on user experience. Overall, minor adjustments were recommended to improve navigation, customization, and user guidance, while the app’s visual and functional design was generally well received [42].

### Ethical Considerations

This study was reviewed and approved by the Rush University Institutional Review Board (approval number 21122902), and the protocol was registered on ClinicalTrials.gov (NCT06359990). Informed e-consent was obtained from all participants. For those under 18 years of age, parental or guardian consent and participant assent were obtained before enrollment. Participants were informed of the voluntary nature of their involvement, their right to withdraw at any time without penalty, and measures to protect their confidentiality and data privacy. All data were deidentified, coded using unique participant IDs, and stored in encrypted, password-protected databases accessible only to authorized study staff. No identifiable information was linked to survey responses or intervention data. Given the vulnerability of this population, additional safeguards were implemented, including private consent procedures, information on local support services, and on-call support for participants reporting distress or imminent risk. To compensate participants for their time and contribution, US $50 was provided after completing the pretest survey and intervention, and an additional US $50 (Target or Amazon e-gift card) was distributed 4 weeks later following completion of the posttest questionnaires.

### Study Procedures

#### Participants: Inclusion and Eligibility Criteria

Eligible participants met the following criteria: (1) male-identifying; (2) Black/African American; (3) aged 15‐24 years; (4) a score >1 on the 4-item Serious Fighting, Friend Weapon Carrying, Community Environment, and Firearm Threats (SaFETy) clinical screening tool, indicating elevated risk for future firearm violence based on 4 domains: Serious Fighting, Friends’ Weapon Carrying, Community Environment, and Firearm Threats [43,44]; (5) English literate; and (6) able to assent/consent (with parental consent if under 18).

Exclusion criteria were (1) being detained or incarcerated; (2) inability to complete assent/consent and assessment forms due to language barriers, cognitive dysfunction, or injury; (3) presenting with an acute psychotic disorder, a suicide attempt as the mechanism of firearm injury, or a recent sexual assault requiring intensive psychosocial support; and (4) a score ≥6 on the SaFETy tool, as such individuals were referred to more intensive programs, as recommended by Goyal et al [45]. Recruitment was successfully conducted in collaboration with community partners, including local organizations, school-based health centers, and social media, with minimal loss to follow-up due to the intervention’s brief and single-session format.

Upon expressing interest in the study, participants completed a sociodemographic enrollment form on Research Electronic Data Capture (REDCap; Vanderbilt University), adapted from the biennial cross-sectional Youth Risk Behavior Surveillance System (YRBS) survey. All study procedures were reviewed and approved by the Rush University Institutional Review Board. Informed e-consent was obtained in accordance with federal and institutional guidelines for research involving minors using REDCap. Consent and assent forms were written in age-appropriate and culturally sensitive language, and all participants were informed of their right to decline or withdraw at any time without penalty.

Participants then completed a pretest survey battery comprising 63 questions assessing psychological distress (Kessler Psychological Distress Scale [K10]), depression (8-item Patient Health Questionnaire [PHQ-8]), attitudes toward guns and violence (Attitudes Toward Guns and Violence Questionnaire [AGVQ]), and aggression (Reactive-Proactive Aggression Questionnaire [RPQ]). All survey questions remained unchanged, as these instruments have been previously validated for use with children and adolescents. To ensure greater control over participant access, engagement tracking, and data capture during the pilot phase, the intervention was delivered via REDCap, with embedded YouTube-hosted videos simulating the app’s video modules. This approach enabled secure and streamlined testing of the core acceptance and commitment therapy (ACT)–based video modules before full app deployment, allowing us to evaluate the acceptability and preliminary effects of the behavioral modules while testing fidelity to content delivery and use styles, and engagement tracking.

Immediately following baseline survey completion, participants were given access to the BrotherlyACT modules and instructed to complete all video-based modules and in-app content either in 1 sitting or at their own pace within a 48-hour window. The posttest survey was administered electronically exactly 4 weeks (28 days) after the pretest, regardless of when participants completed the intervention content.

Additionally, we tracked the amount of time participants spent on the REDCap page and their engagement with the videos (ie, in-app quiz completion, likes and comments). Participants who completed the video content prematurely, either by not viewing all the materials or by skipping through them rapidly, were encouraged to reengage through various means, including indicating approval, providing comments, or submitting a summary of the knowledge acquired from the video content via email. Incentives for participating in the study were distributed after completion of the pretest and video modules (US $50) and again 4 weeks later, following completion of the posttest questionnaires (US $50 in the form of Target and Amazon e-gift cards).

#### Intervention: BrotherlyACT’s Video-Based Psychoeducational Modules

Drawing from evidence-based behavior change models and community insights, the app incorporated elements that address the personal, social, and environmental factors contributing to youth firearm-related violence. Its development involved collaborative design with young Black males from the target age group, ensuring that the app’s content, imagery, language, and delivery methods were culturally attuned to their real-life experiences.

As at testing, the app comprised a set of 8 concise, video-based psychoeducational modules that depict realistic situations. These modules address topics such as refusing guns, resisting gang involvement, practicing gun safety, saying no to alcohol and drugs, resolving conflicts, avoiding retaliation, recognizing healthy and unhealthy relationships, practicing mindfulness, cultivating a positive outlook on the future, and using adaptive coping strategies. They incorporate behavioral skills training, emotional regulation techniques, and decision-making strategies to help reduce youth violence and risky firearm behavior. The app’s video modules are primarily based on ACT [46-49], a third-wave cognitive behavioral therapy that emphasizes accepting and navigating difficult thoughts and emotions rather than attempting to persistently change or avoid them [50-54]. ACT has demonstrated efficacy in improving emotional regulation and reducing aggression among trauma-exposed youth, who often cannot control their environments but can control how they respond to and navigate these chronic and intersecting issues [55]. Utilizing a micro-learning approach, complex topics were broken down into smaller, ready-to-use and life skill-based components to enhance comprehension, application, and retention, particularly for youth with various attention spans [56,57].

A Black male narrator presents the modules with multimedia and interactive heuristics, offering skills for navigating and preventing firearm-related scenarios such as peer pressure to carry weapons, retaliatory violence, and rejecting gang involvement. The modules use culturally familiar language and visuals, with a tone that emphasizes respect, empowerment, motivational enhancement, and nonpunitive language to avoid pathologizing young people’s behavior. Table 1 summarizes the behavioral skill objectives and theoretical frameworks underpinning each BrotherlyACT module.

**Table 1.** BrotherlyACT module overview: focus areas, behavioral skills, and theoretical underpinnings.

Module title	Module target	Behavioral skills taught	Theoretical underpinning
Gun Safety 101	Firearm safety and harm reduction	Safe storage awareness and noncarry decision-making	Harm reduction theory and social cognitive theory
Gun Refusal Skills 101	Peer pressure and firearm refusal	Assertive communication and verbal refusal strategies	Social norms theory and motivational interviewing
Healthy Relationship Skills	Intimate partner and peer conflict	Emotional literacy, boundary-setting, and consent communication	Attachment theory and acceptance and commitment therapy
Gang Resistance Skills	Gang avoidance and safety navigation	Risk recognition, exit planning, and identity affirmation	Strength-based approach and narrative therapy
Coping Skills	Managing stress, trauma, and emotion	Emotion regulation, distress tolerance, and adaptive self-talk	Acceptance and commitment therapy and cognitive behavioral theory
Mindfulness for Black Boys	Emotional regulation and trauma recovery	Grounding, breathwork, and body scanning	Acceptance and commitment therapy and mindfulness-based stress reduction
Alcohol and Drug Refusal Skills	Substance use prevention	Refusal techniques and decision-making	Social learning theory and motivational interviewing
Conflict Resolution Skills	De-escalation and self-regulation	“Cool down” tools, perspective-taking, and problem-solving	Acceptance and commitment therapy and problem-solving therapy

The broader BrotherlyACT app also includes additional features such as (1) a safety planning toolkit with interactive tools for mood tracking, risk assessment, mindfulness exercises, goal setting, and a zip code–enabled directory of local mental health services, community support resources, and violence prevention programs within a 50-mile (80.47 km) radius; and (2) service engagement and talk therapy via an artificial intelligence–powered chatbot named DEVON, a confidential and programmed resource for discussing stressors, emotional triggers, and challenges related to violence and substance use. DEVON was designed as a trauma-informed conversational agent to simulate peer dialogue and support help-seeking behaviors. Embedded within the app interface, the chatbot provides confidential, just-in-time emotional support accessible outside structured intervention sessions. Conversations with DEVON are anonymous, with user input logged only as anonymized session data. Participants are advised not to enter personal identifiers. App access was password-protected and intended for use on personal or study devices in private settings. These additional features were not evaluated in this study.

### Sociodemographic Measures

#### Overview

We adapted items from the Centers for Disease Control and Prevention’s biennial YRBS, which assesses health-related behaviors among a nationally representative sample of US students in grades 9‐12. Our analysis included sociodemographic variables such as age, gender, race/ethnicity, socioeconomic status (ie, household income), school and employment status, zip code or address, dating relationship status, and smartphone ownership. The questionnaire also assessed violence (physical and psychological violence toward a partner, 30-day weapon carriage, and community violence) and substance use experiences (ie, 30-day adolescent binge drinking, nonmedical drug use, and substance-related violence perpetration). Substance use behaviors were assessed due to their known association with youth aggression, trauma exposure, and risky weapon-related behaviors [8,27,58]. These variables were included to examine participants’ broader behavioral profiles and identify overlapping intervention targets. Table 2 summarizes the sociodemographic characteristics of participants who completed the pretest and posttest questionnaires and received the intervention (ie, the analytic sample).

**Table 2.** Sociodemographic characteristics of study participants.

Sociodemographic variables and descriptors	Sample, n (%)
Age (years)^a^	
15‐18	11 (16)
19‐24	58 (84)
Ethnicity	
Hispanic/Latinx	7 (10)
Non-Hispanic	63 (90)
Education level	
Some high school (eg, general educational development)	13 (19)
High school diploma	20 (29)
Some trade/technical school	11 (16)
Some college or university	24 (34)
Master’s degree	1 (1)
Other	1 (1)
Relationship status	
Dating (serious, casual, or hooking up)	30 (43)
Single	39 (56)
Common law: living with partner/significant other	1 (1)
Employment status^b^	
Full-time (working at least 35 hours/week)	9 (13)
Part-time (working less than 35 hours/week)	34 (49)
Laid off or unemployed	14 (20)
Student	12 (17)
Other	1 (1)
Total annual household income	
Less than US $20,000	17 (24)
US $20,000-US $39,999	17 (24)
US $40,000-US $59,999	15 (21)
US $60,000-US $79,999	11 (16)
US $80,000-US $99,999	7 (10)
US $100,000 or more	3 (4)
Binge drinking in the past 30 days: During the past 30 days, what is the highest number of alcoholic drinks you had in a row, that is, within a couple of hours?	
0‐0 drinks	17 (24)
1‐1 or 2 drinks	5 (7)
2‐3 drinks	15 (21)
3‐4 drinks	16 (23)
4‐5 drinks	11 (16)
5‐6 or 7 drinks	2 (3)
6‐8 or 9 drinks	3 (4)
7‐10 or more drinks	1 (1)
Illicit drug use: Used any medications or drugs other than those required for medical reasons	
No	31 (44)
Yes	39 (56)
Violence after substance use: Have you been involved in, perpetrated, or committed any sort of violence or aggression after taking any drugs or alcohol?	
No	38 (54)
Yes	32 (46)
Serious physical fight (past 6 months)	
0 (never)	22 (31)
1 (once)	11 (16)
2 (twice)	17 (24)
3 (3‐5 times)	19 (27)
4 (6 or more times)	1 (1)
Friend who carried weapons in the past 6 months: How many of your friends have carried a knife, razor, or gun^c^?	
1 (none)	31 (45)
2 (some)	31 (45)
3+ (many, most, or all)	7 (10)
Threatened with a gun in the past 6 months: How often, in the past 6 months, including today, has someone pulled a gun on you?	
0 (never)	31 (44)
1 (once)	22 (31)
2+ (twice or more)	17 (24)
Heard gunshots in the past 6 months: In the past 6 months, how often have you heard guns being shot?^d^	
0 (never)	5 (7)
1 (once or twice)	8 (12)
2 (a few times)	30 (44)
3 (many times)	25 (37)
Community violence in the past year: Have you been impacted by any sort of violence or aggression in the last 12 months (eg, physical fighting, dating or relationship violence, school violence, bullying, threats with weapons, and gang-related violence)?	
Yes	53 (76)
No	17 (24)
Have you ever seen someone get physically attacked, beaten, stabbed, or shot in your community or neighborhood?	
No	13 (19)
Yes	57 (81)
Psychological violence toward partner in the past year: In the past year, have you been psychologically aggressive toward a romantic or dating partner (eg, threatened to hurt them, insulted them, spoke to them in a hostile or mean tone of voice, threatened them with a weapon or threatened to hit them or throw something at them)?	
No	29 (41)
Yes	41 (59)
Physical violence toward partner in the past year: In the past year, have you been physically aggressive toward a romantic or dating partner (eg, slapped them, kicked, hit, punched them, or threatened them with a weapon)?	
No	39 (56)
Yes	31 (44)
Carried a weapon in the past 30 days: During the past 30 days, how many days did you carry a weapon such as a gun, knife, or club?	
0 days	33 (47)
1 day	9 (13)
2 or 3 days	17 (24)
4 or 5 days	9 (13)
4‐6 or more days	2 (3)

a Age missing for 1 participant; percentages calculated among respondents to this item (n=69).

b Employment status categories are not mutually exclusive; respondents could select more than 1 type (eg, part-time worker and student). Counts therefore exceed N. Percentages are calculated using the total sample size (N=70).

c Item nonresponse for 1 participant; percentages calculated among respondents to this item (n=69).

d Item nonresponse for 2 participants; percentages calculated among respondents to this item (n=68).

#### The SaFETy Score

The SaFETy score, a 4-item firearm risk assessment questionnaire included in our enrollment survey, was used to categorize participants into low-risk (0), medium-risk (1-5), and high-risk (≥6) groups for firearm violence based on weighted item responses. The questionnaire inquired about the frequency of serious physical fights, friends carrying weapons, hearing gunshots, and having guns pulled on them in the past 6 months. This clinically feasible tool has been shown to correlate with firearm violence, although further validation is needed [43]. The SaFETy score demonstrates potential in predicting future firearm violence risk, with each point increase associated with a 1.5-fold higher likelihood of firearm violence within 2 years (odds ratio 1.5), underscoring its value as a clinical screening instrument [44,59]. Administration of the tool takes less than 1 minute, and Goldstick et al [44] suggested referring patients with scores ≥6 to more intensive programs.

#### Attitudes Toward Guns and Violence

The 23-item AGVQ assesses individual attitudes toward using guns and violence for conflict resolution, power assertion, or safety across 4 scales: Aggressive Response to Shame (disrespect sensitivity; items 1, 2, 5, 10, 15, 16, 18, and 20), Comfort With Aggression (violence acceptance; items 8, 12, 13, 17, 21, and 22), Excitement (gun-related excitement; items 6, 7, 9, 11, and 14), and Power/Safety (power and safety from guns; items 3, 4, 19, and 23). Cronbach α coefficients were 0.83, 0.81, 0.79, and 0.72, respectively. Respondents rated their agreement on a scale from 0 (disagree) to 2 (agree). The summed scores for the AGVQ subscales ranged from 0 to 46, with higher scores indicating more favorable attitudes toward guns and violence. Juvenile firearm possession is linked to violence lethality and may reflect risky exposure to firearms or a proviolence viewpoint. Although the correlation between attitude and behavior change varies, modifying attitudes is crucial for changing behavior [60]. Youth typically complete the AGVQ in 5‐10 minutes. The AGVQ correlates with handgun ownership [31] and with various psychological and behavioral assessment instruments, supporting its construct validity and helping to understand how attitudes toward guns and violence relate to broader patterns of violent behavior, cognitions, and emotions. Although the AGVQ remains an insufficiently addressed aspect of pediatric firearm violence prevention, interventions such as the “Cradle to Grave” program have shown potential in reducing AGVQ scores, particularly among students with higher baseline tendencies toward violence [61].

#### The Reactive-Proactive Aggression Questionnaire

The RPQ is a 23-item validated self-report instrument designed to differentiate between reactive aggression (11 items; eg, “Reacted angrily when provoked by others”) and proactive aggression (12 items; eg, “Had fights to show who was on top”). Respondents reflect on these behaviors over the past 30 days, rating each item on a 3-point Likert scale (0=never, 1=sometimes, and 2=often). Subscale scores were summed to generate separate reactive and proactive aggression scores, as well as a combined total aggression score. Higher scores indicate a greater frequency of aggressive behaviors [29,62]. The RPQ has demonstrated good reliability and construct validity in adolescent populations and has been extensively used in research on aggression, violence, and trauma exposure. Reactive aggression is more strongly linked to internalizing symptoms, such as anxiety and depression, whereas proactive aggression is associated with externalizing and antisocial traits [29,62].

#### Kessler Psychological Distress Scale

The K10 [63] is a widely used self-report diagnostic tool for identifying individuals who may require further evaluation for anxiety and depression. It is applicable in both the general population and clinical settings. The K10 comprises 10 questions rated on a 5-point scale (5=all the time to 1=none of the time), reflecting experiences over the past 4 weeks. Scores range from 10 (no distress) to 50 (severe distress), with lower scores indicating less distress. The scale includes 2 subscales: depression (items 1, 4, 7, 8, 9, and 10) and anxiety (items 2, 3, 5, and 6). Items 3 and 6 are omitted if the previous response is “none of the time” [64].

#### Patient Health Questionnaire-8

The PHQ-8 is extensively used to evaluate depression severity in clinical and research settings [65,66]. It comprises 8 items aligned with the Diagnostic and Statistical Manual of Mental Disorders, Fifth Edition criteria for major depressive disorder and serves as a reliable tool for screening and monitoring depressive symptoms. The PHQ-8 is a shortened version of the PHQ-9, omitting the item on suicidal ideation. It assesses mood and feelings over the past 2 weeks on a 4-point scale from 0 (not at all) to 3 (nearly every day), with total scores ranging from 0 to 24. Scores above 10 indicate moderate depression, while scores above 20 indicate severe major depression.

### Data Analysis

All analyses were conducted using the JASP software system (version 0.18.3 for Windows) and Microsoft Excel, with results subsequently verified using STATA (version 18; StataCorp LLC). Participant characteristics are presented as means with SDs for continuous variables, and as frequencies and percentages (%) for categorical variables.

All surveys were scored to calculate total and subscale raw scores. Higher scores indicated a proclivity toward violence or aggression (AGVQ and RPQ) or poorer mental health status (K10 and PHQ-8). Paired-sample *t* tests and Wilcoxon signed rank test (a nonparametric measure) were used to detect differences in scores across all validated instruments, with *P* values <.05 (2-tailed hypothesis) considered statistically significant.

Initial data analysis focused on ensuring data quality by checking for allowable ranges, errors, and patterns of missing data, as well as examining outliers and variable distributions to confirm alignment with the assumptions underlying the planned analyses. Missing data were observed in 6 out of 70 (9%) cases and ≤3-4 out of 70 (<5%) variables. This missingness was addressed using listwise deletion, which may have introduced bias if the data were not missing completely at random. While this approach preserved the internal consistency of the paired analyses, it may limit the generalizability of the findings and reduce statistical power.

Owing to limited prior effect size estimates, we conducted an a priori power analysis using G*Power 3.1.9.7 to determine the minimum sample size required to detect a medium effect (*dz*=0.5) in a paired-sample *t* test comparing pre- and postintervention scores on the AGVQ. Assuming α=.05 and 95% power, the minimum required sample size was 54 participants. To account for potential attrition and up to 20% missing posttest data, we oversampled and enrolled 70 young Black males (aged 15‐24 years) from a larger multiphase study (N=312). Pre- and postintervention data were analyzed to evaluate statistically significant changes and effect sizes across key outcomes.

## Results

### Participant Characteristics

Our sample consisted of 70 young Black men aged 15‐24 years (mean age 20.97, SD 2.44 years). All participants identified as African American, and 7 (10%) also identified as Hispanic or Latinx. Educational attainment varied, with 18 (26%) having less than or equal to a high school diploma, and 22 (31%) reporting a college or university education. Nearly half of the participants (34/70, 49%) worked part-time (<35 hours per week). Over half were single (39/70, 56%) or dating (30/70, 43%). Smartphone ownership was nearly universal (68/70, 97%), highlighting access to digital intervention. Regarding annual household income, 46 (66%) reported between US $40,000 and US $59,999. Most participants were recruited from Illinois (47/70, 67%), followed by Florida (6/70, 9%), New York (4/70, 5%), Texas (3/70, 4%), and Pennsylvania (3/70, 4%). Other participants were recruited from Alabama, Georgia, Michigan, New Jersey, Louisiana, and Wisconsin.

### Firearm Violence Profile

To characterize the participants’ violence exposure and firearm risk, we collated both descriptive data (ie, YBRS survey questions) and domain-specific scores (ie, SaFETy screening tool).

### Descriptive Profile of Violence Exposure

#### Community Violence and Interpersonal Aggression: Participant Experiences

Nearly half of the participants (32/70, 46%) reported engaging in a serious physical fight within the past 6 months. During the same period, 31 (44%) indicated that a friend had carried a weapon (knife, razor, or gun), and 31 (44%) reported having been directly threatened with a gun at least once. Additionally, 41 (59%) had heard gunshots in their neighborhood in the previous 6 months. Exposure to community violence was highly prevalent, with 49 (70%) participants endorsing direct or indirect involvement in violence (eg, fighting, threats, or gang activity) in the past year. Furthermore, 53 (76%) respondents reported witnessing someone being attacked, beaten, stabbed, or shot in their neighborhood. Regarding relationship aggression, 41 (59%) reported psychological aggression toward a romantic or dating partner, and 31 (44%) disclosed having engaged in physical aggression in the past year. Notably, 31 (44%) reported carrying a weapon at least once in the past 30 days.

#### SaFETy Score Breakdown

Table 3 presents item-level responses and scoring contributions for each domain of the SaFETy score: Serious Fighting, Friend Carrying Weapons, Community Environment, and Firearm Threats. The most commonly endorsed level for Serious Fighting was “3‐5 times in the past 6 months,” reported by 19 of 70 (27%) participants, contributing 2 points to the SaFETy score. In the Friend Carrying Weapons domain, 38 (54%) participants reported that at least some of their friends had carried a weapon in the past 6 months, with 7 (10%) participants reporting that many, most, or all of their friends had done so, contributing 1 point.

**Table 3.** Breakdown of SaFETy^a^ score distribution.

Category and question/scale levels	Values, n (%)	SaFETy contribution
Serious Fighting		
In the past 6 months, including today, how often did you get into a serious physical fight?		
0 (never)	22 (31)	0
1 (once)	11 (16)	1
2 (twice)	17 (24)	1
3 (3‐5 times)	19 (27)	1
4+ (6 or more times)	1 (1)	4
Friend Carrying Weapons		
In the past 6 months, including today, how many of your friends have carried a knife, razor, or gun?		
1 (none)	31 (44)	0
2 (some)	31 (44)	0
3+ (many, most, or all)	7 (10)	1
Community Environment		
In the past 6 months, how often have you heard guns being shot?		
0 (never)	5 (7)	0
1 (once or twice)	8 (11)	0
2 (a few times)	30 (43)	0
3 (many times)	25 (36)	1
Firearm Threats		
How often, in the past 6 months, including today, has someone pulled a gun on you?		
0 (never)	31 (44)	0
1 (once)	22 (31)	3
2+ (twice or more)	17 (24)	4

a SaFETy: Serious Fighting, Friend Weapon Carrying, Community Environment, and Firearm Threats.

Community environment exposure was notably high: 57 (81%) participants reported hearing gunshots at least “a few times” in the past 6 months, with 26 (37%) hearing them “many times,” contributing 3 points. The highest-weighted item was Firearm Threats: 17 (24%) participants reported being threatened with a gun 2 or more times, contributing 4 points. Notably, 22 (31%) respondents scored 0 across all categories of firearm threat. These distributions reflect a high prevalence of direct and indirect exposure to firearm violence in this sample, contributing to higher cumulative SaFETy scores and elevated future firearm violence risk.

The distribution of total SaFETy scores among participants reveals a wide range of firearm risk exposure. While a notable portion of the sample scored 0, indicating minimal or no reported exposure to serious fighting, weapon-carrying peers, gunfire in the community, or direct firearm threats, the majority reported at least one source of exposure. A significant subset scored between 4 and 7, reflecting high cumulative exposure, particularly in the domains of community gun violence and direct firearm threats. This right-skewed distribution suggests heterogeneity in risk profiles, with a sizable group facing elevated vulnerability to firearm-related harm, an important consideration for intervention targeting and future risk stratification.

### Substance Use Profile

We measured 30-day adolescent binge drinking, nonmedical drug use, and substance-related violence perpetration. Nearly one-fourth of the participants (17/70, 24%) had engaged in binge alcohol consumption, defined as more than 5 drinks in a single sitting within the past 30 days. Over half reported illicit use of pharmaceuticals or narcotics for nonmedical purposes, impacting 39 (56%) participants. Finally, 32 (46%) participants reported perpetrating substance-related violence.

### Intervention Preliminary Effects

#### Overview

We assessed multiple outcomes (summarized in Table 4), such as attitudes toward guns and violence, reactive and proactive aggression, and mental health outcomes (eg, psychological distress).

**Table 4.** Pretest and posttest changes in the study outcomes.^a^

Outcome	Test used	Pretest, mean (SD)	Posttest mean (SD)	*t* test (*df*) or *z *value	*P* value	Effect size (Cohen *d*)
Attitudes toward guns and violence	Paired *t* test	29.8 (8.7)	26.1 (9.0)	3.52 (44)	<.001	0.53
Reactive aggression	Paired *t* test	10.48 (5.39)	8.67 (4.92)	2.74 (53)	.008	0.37
Proactive aggression	Paired *t* test	6.89 (4.95)	6.70 (5.01)	0.82 (53)	.42	0.11
Psychological distress (Kessler Psychological Distress Scale)	Paired *t* test	18.2 (6.3)	18.1 (6.2)	0.20 (58)	.84	0.03
Depressive symptoms (8-item Patient Health Questionnaire)	Wilcoxon signed rank test	9.82 (6.41)	9.33 (6.26)	0.90^b^	.37	N/A

a *P*<.05 considered statistically significant. Paired *t* tests were used for normally distributed outcomes, and the Wilcoxon signed rank test was used for nonnormally distributed data (8-item Patient and Health Questionnaire).

b *z* value.

#### Attitudes Toward Guns and Violence

A significant reduction in AGVQ scores was observed among participants (n=43) from pretest (mean 29.8, SD 8.7) to posttest (mean 26.1, SD 9.0), with a mean difference of 3.69 (SE 1.05, *t*_42_=3.522, *P*<.001), and a moderate effect size (Cohen *d*=0.53). Subscale analysis revealed the most considerable improvement in the “Aggressive Response to Shame” subscale (28% reduction), followed by the “Excitement Toward Guns and Violence” subscale (14.8%), the “Feelings of Power/Safety” subscale (8%), and the “Comfort with Aggression” subscale (2.3%). These crucial findings indicate that participants experienced reduced emotional reactivity to perceived threats and decreased excitement and reliance on firearms for power and safety. For example, Goldberg et al [61] found that small but statistically significant decreases in AGVQ scores were predictive of lower intentions to carry firearms and decreased willingness to use violence in future peer conflict situations.

#### Reactive and Proactive Aggression

Mean scores indicated that reactive aggression significantly decreased from 10.48 (SD 5.39) preintervention to 8.67 (SD 4.92) postintervention (mean difference 1.82, SE 0.66, *t*_53_=2.74, *P*=.008), with a small-to-moderate effect size (Cohen *d*=0.37), suggesting a notable improvement in managing impulsive, emotionally driven responses to perceived threats following exposure to the intervention. By contrast, no significant change was detected in proactive aggression scores between the pre- and posttest (*t*_53_=0.82, *P*=.42), with a small effect size (Cohen *d*=0.11), indicating the need for longer-term or more intensive and targeted interventions to address the underlying cognitive patterns and motivations associated with planned aggression.

#### Psychological Distress

A paired-samples *t* test was used to evaluate changes in psychological distress, focusing on subscales (depression and anxiety) and overall psychological distress (ie, total scores) from pre- to posttest. No significant changes were found in depression symptoms from pretest (mean 15.85, SD 6.28) to posttest (mean 15.97, SD 5.85, *t*_58_=–0.20, *P*=.84, Cohen *d*=0.03). Likewise, anxiety scores did not significantly differ between pretest (mean 10.32, SD 3.93) and posttest (mean 10.02, SD 3.88, *t*_58_=0.76, *P*=.45, *d*=0.10). In addition, overall psychological distress scores remained stable from pretest (mean 26.17, SD 10.02) to posttest (mean 25.98, SD 9.54, *t*_58_=0.20, *P*=.84), with a negligible effect size (*d*=0.03). At baseline, 21 of 70 (30%) respondents reported potentially moderate to severe psychological distress (K10 scores>16), highlighting a substantial mental health burden within this sample.

A change of 5 points or more on the K10 is considered clinically meaningful for psychological distress [67,68]. Based on our sample mean difference (mean difference 0.186), the absence of both statistically and clinically significant changes suggests that the intervention may have had a limited short-term impact on psychological distress, underscoring the need for enhanced or sustained mental health support in the long term.

#### Depression (PHQ-8)

The Shapiro-Wilk test revealed a significant deviation from normality in the pretest scores (*W*=0.939, *P*=.008), indicating a need for a nonparametric approach. A Wilcoxon signed-rank test was conducted to compare pretest (mean 9.82, SD 6.41) and posttest (mean 9.33, SD 6.26) scores. The results showed no statistically significant difference (*W*=597.50, *z*=0.903, *P*=.37) in depression, corroborating earlier findings. Although descriptive statistics suggested a slight reduction in the mean PHQ-8 scores, this change was not significant. For the PHQ-8, a reduction of 5 points or more is widely accepted as the minimal clinically important difference in both primary care and research settings [66].

#### Intervention Engagement Outcomes

Backend analytics from REDCap were used to monitor time stamps and session durations. Almost all participants (68/70, 97%) completed the video modules in a single session, with 47 (67%) finishing within an hour, suggesting high feasibility of BrotherlyACT as a digital intervention. Participants were advised to complete 1-2 modules daily, with no upper limit per session. This flexible approach accommodated varying schedules and comfort levels while ensuring consistent content delivery. Future iterations will aim to explore sustained engagement and assess feasibility in school and clinical settings.

## Discussion

### Principal Findings

This study evaluated the preliminary effects of BrotherlyACT, a culturally responsive, trauma-informed, multicomponent mobile and online intervention designed to support young Black males (aged 15‐24) in navigating and preventing community violence, substance use, and mental health challenges. The app aims to improve access to precrisis support and mental health resources for youth living in low-resource, high-violence environments. BrotherlyACT builds on promising ED-based models, such as that by Carter et al [17], by expanding intervention reach beyond clinical settings. Its mobile delivery, cultural relevance, and trauma-informed framework address key barriers to care—namely, limited access to early interventions, lack of continuity, and low trust in traditional systems.

Our findings demonstrated a significant reduction in overall attitudes toward guns and violence, with the most considerable reduction occurring in the “Aggressive Response to Shame” subscale (a 28% decrease). This result aligns with research linking shame and emotional dysregulation to aggression, particularly in populations exposed to high levels of systemic inequities and chronic community violence [55,69,70]. Shame proneness during adolescence is a significant psychological construct linked to aggressive behavior, often mediated by hostility and externalization of blame [71].

Additionally, notable reductions in “Excitement Toward Guns and Violence” (14.8%) and “Feelings of Power/Safety” (8%) suggest meaningful shifts in attitudes associated with the perceived role of firearms in self-protection, empowerment, and identity formation among adolescents. These changes reflect progress in addressing key psychosocial drivers of risky firearm behavior. The medium effect size for the overall AGVQ outcome (*d*=0.53) also suggests that the observed difference was not only statistically significant but also practically notable. We posit that the intervention effectively shifted attitudes toward firearms and violence by challenging norms that reinforce gun carrying and retaliation, and by altering cognitive frameworks around violence, likely through the promotion of nonviolent problem-solving and prosocial coping strategies. These attitudinal changes may reflect the malleability of belief systems when disrupted by relatable, peer-aligned narratives. The app’s nondidactic tone and culturally resonant scenarios may have reduced defensiveness and increased engagement, particularly among youth resistant to adult-led violence messaging.

In terms of aggressive behavior, the intervention’s differential impact on the *reactive aggression* subtype underscores its potential to mitigate impulsive, emotionally driven aggression, a key contributor to firearm injuries and homicides in urban settings [72]. Several mechanisms may explain these shifts. First, the app’s video content was designed to engage users through culturally salient and emotionally resonant messaging, aligning with principles of social learning theory and narrative persuasion frameworks. Youth may have identified with the characters and scenarios presented, leading to emotional mirroring and internalization of the modeled prosocial behaviors. Notably, reactive aggression, typically driven by perceived threat and emotional dysregulation, may have been mitigated through the vicarious modeling of alternative conflict resolution strategies presented in the modules.

Conversely, we observed a nonsignificant decline in proactive aggression, which prior studies suggest may be more responsive to interventions that explicitly target cognitive processes such as empathy and moral reasoning [73,74]. Addressing proactive aggression may also require longer-term interventions, such as dialectic behavioral therapy, in combination with content that affirms identity, fosters critical consciousness, challenges harmful masculinity norms, and promotes consequential thinking and value realignment. Importantly, interventions targeting proactive aggression must also address the broader social determinants of health that shape youth behavior.

Similarly, no significant changes were observed in psychological distress (depression or anxiety), aligning with prior research in the neuroscience of aggression that underscores the importance of trauma-informed or mental health–focused components—approaches shown to be effective in analogous nondigital contexts [75]. This finding was anticipated, as key mental health features of BrotherlyACT, including the safety planning toolkit and artificial intelligence–powered talk therapy chatbot, were not fully implemented or tested at this stage. Additionally, it remains unclear whether the minimal changes observed in depression were cognitive, affective, or somatic in nature, an area that will be explored in the larger planned study.

The absence of change in psychological distress supports the notion that distress may not be the primary mechanism through which video-based interventions reduce aggression. However, we are investigating a hypothesized pathway of effects in supplemental analysis. Nonetheless, these findings provide a robust foundation for refining intervention strategies aimed at improving both aggression-related outcomes and mental health outcomes.

Lastly, the significant rate of substance misuse in this group emphasizes the critical need to cotarget drug and alcohol use as a concurrent risk factor within this population. Over half of the participants (39/70, 56%) reported illicit drug use, and substance-related violence perpetration (32/70, 46%). These findings are consistent with other studies linking substance use to elevated risks of both perpetrating and experiencing firearm violence [58,76]. Future interventions may benefit from expanding current modules that directly address substance misuse, given its interplay with firearm violence.

### Implications for Practice and Prevention

Traditional firearm safety interventions for youth often emphasize parent-focused messaging and home-based storage practices. However, these strategies may not align with how at-risk older adolescents, particularly young Black males, access and engage with firearms. Such approaches frequently overlook structural realities, such as neighborhood-level firearm acquisition, systemic mistrust of support systems, and limited access to secure and inexpensive storage options. BrotherlyACT addresses these gaps by directly engaging high-risk youth through developmentally appropriate, culturally grounded modules focused on gun safety, nonretaliation, and coping within violence-impacted environments.

Critically, the intervention is grounded in trauma-informed design and rooted in evidence-based behavioral frameworks, including ACT and motivational interviewing. These frameworks promote emotional regulation, values-driven decision-making, and self-reflection, offering a nonstigmatizing, empowering alternative to punitive behavior change models. Given that 97% of US adolescents own smartphones [77], BrotherlyACT offers a scalable and discreet solution for reaching youth who may avoid traditional services. The high levels of engagement observed in this study underscore its feasibility; however, variability in module completion points to the need for additional engagement strategies, such as personalized incentives or enhanced interactive features to improve module completion and reuse.

Reducing firearm-related injuries and deaths among young Black males requires a multilevel public health strategy. This includes individual behavioral interventions, community-level engagement, and systemic policy changes, all underpinned by trauma-informed, culturally sensitive approaches, including direct-to-youth interventions that address care barriers and societal drivers of firearm violence [3,78]. Ultimately, BrotherlyACT contributes a novel, culturally responsive tool to this effort. Future research should explore how digital tools such as BrotherlyACT can integrate with broader structural and environmental interventions to produce sustainable, equity-centered outcomes.

Because of its modular design and digital delivery, BrotherlyACT is particularly well-suited for integration into school-based health centers, community mental health clinics, after-school programs, and youth-serving community-based organizations. Importantly, the intervention is consistent with both citywide and national priorities that emphasize trauma-informed, equity-centered approaches to the prevention of youth gun violence. For instance, in Chicago, we are investigating opportunities to align BrotherlyACT with the city’s *Office of Gun Violence Prevention* and its trauma recovery ecosystem, as well as with initiatives funded under the Chicago Youth Service Corps.

At the policy level, this study provides preliminary evidence to inform future digital health innovation grants, Medicaid reimbursable pilots for technology-based behavioral health tools, and public health department efforts aimed at expanding digital care access for underserved youth. Our proposed scale-up model includes collaboration with public school systems, local Federally Qualified Health Centers, and youth justice diversion programs, guided by implementation science frameworks and equity-based partnership principles. Future research will focus on formalizing these implementation pathways, evaluating institutional readiness, and identifying sustainable policy mechanisms for long-term adoption.

### Limitations and Strengths

A central strength of this study is its focus on an urban, high-risk sample of young Black males most impacted by firearm violence. The utilization of validated self-report instruments, namely, the AGVQ and RPQ, facilitated a direct assessment of attitudes and behaviors related to aggression, specifically in the context of gun violence. Previous research corroborates the reliability of these measures within similar populations [79]. Furthermore, the confidential and computerized format of our survey likely mitigated response bias concerning sensitive topics such as gun use and substance involvement, particularly in a population that exhibits a high level of mistrust regarding the disclosure of personal information and details.

However, some limitations are worth noting. Self-report data remain vulnerable to social desirability bias, which may have influenced participants’ disclosures. While this could inflate observed changes, it is a common limitation in violence prevention research. Additionally, the RPQ and AGVQ may oversimplify the complex and context-dependent nature of youth attitudes toward firearms and aggression. Although we reported subscale results where appropriate, aggregating scores can obscure important variability. In addition, the pilot design was not powered to detect subgroup differences or interaction effects. While missing data were minimal, our use of listwise deletion may have reduced power and introduced bias. Future studies will need to incorporate more robust missing data techniques, such as multiple imputation.

Critically, participants only engaged with the video-based module component of BrotherlyACT via REDCap, rather than the full app interface. This limited exposure may have underestimated the intervention’s true engagement potential. Moreover, we did not capture objective metrics of video engagement (eg, watch time or skipping behavior), limiting interpretation of intervention dose effects. We also did not collect data on firearm use motivations, such as distinctions between recreational, protective, or aggressive carrying, which bear implications for study interpretation. Despite these limitations, module completion rates remained high and nearly all participants completed the content in a single sitting, supporting the feasibility and acceptability of brief digital violence prevention tools. These early findings reinforce the promise of BrotherlyACT as a scalable, youth-centered intervention.

### Conclusions

This study demonstrates the promise of BrotherlyACT as a culturally responsive, trauma-informed tool for addressing both attitudinal and behavioral risk factors linked to firearm violence among young Black males. By targeting reactive aggression and shifting norms around violence, BrotherlyACT offers a novel, scalable approach to primary prevention in medium- to high-risk groups. These findings lay important groundwork for future strategies aimed at reducing dynamic and context-dependent risks, such as seasonal or escalating violence, through brief, targeted interventions. However, given the multifaceted nature of aggression and the systemic barriers faced by this population, further refinement of intervention components and deeper engagement with structural determinants will be critical. Sustained progress in firearm violence prevention will require integrative solutions that blend individual-level change with community and policy-level action to promote long-term safety and well-being.

## Supplementary material

10.2196/70048 Checklist 1 CONSORT-EHEALTH (Consolidated Standards of Reporting Trials of Electronic and Mobile Health Applications and Online Telehealth) checklist.

## Abbreviations

ACT: acceptance and commitment therapy
AGVQ: Attitudes Toward Guns and Violence Questionnaire
CONSORT-EHEALTH: Consolidated Standards of Reporting Trials of Electronic and Mobile Health Applications and Online Telehealth
ED: emergency department
K10: Kessler Psychological Distress Scale
mHealth: mobile health
PHQ-8: 8-item Patient and Health Questionnaire
RPQ: Reactive-Proactive Aggression Questionnaire
SaFETy: Serious Fighting, Friend Weapon Carrying, Community Environment, and Firearm Threats
YRBS: Youth Risk Behavior Surveillance System
